# Research Advancement in Grassland Ecosystem Vulnerability and Ecological Resilience and Its Inspiration for Improving Grassland Ecosystem Services in the Karst Desertification Control

**DOI:** 10.3390/plants11101290

**Published:** 2022-05-11

**Authors:** Jinzhong Fang, Kangning Xiong, Yongkuan Chi, Shuzhen Song, Cheng He, Shuyu He

**Affiliations:** 1 School of Karst Science, Guizhou Normal University, Guiyang 550001, China; fjinz1314@163.com (J.F.); hebeichiyongkuan@163.com (Y.C.); shuzhensong@yeah.net (S.S.); hecheng25@163.com (C.H.); heshuyu13550048382@163.com (S.H.); 2 State Engineering Technology Institute for Karst Desertification Control of China, 116 Baoshan North Road, Guiyang 550001, China

**Keywords:** grassland, ecosystem vulnerability, ecological resilience, ecosystem service, karst desertification control

## Abstract

Karst desertification control of grasslands balances the ecological and economic benefits of ecological restoration and rural ecological animal husbandry development. In the context of global changes and intensified human activities, the fragility of grassland ecosystems under karst desertification control is becoming increasingly evident, and enhancing the ecological resilience and ecosystem services of grasslands is an issue that urgently needs to be addressed. In this paper, the CNKI literature, WOS core databases and Goolgle scholar were used as search sources, identifying 179 articles related to the study of grassland ecosystem vulnerability and ecological resilience. This research systematically reviewed the progress of grassland ecosystem vulnerability research and analyzed the relationship between grassland ecosystem services (GESs) and grassland ecosystem vulnerability and resilience. The direction of enhancing GESs in karst areas is indicated in terms of the reciprocal feedback, synergistic relationship, and mechanism of action of GESs, vulnerability, and resilience. It is also emphasized that the karst desertification area should provide an ecological foundation for the sustainable development of the regional environment around the supply-and-demand relationship of GESs, the trade-off synergy of service flow, and the enhancement of ecological resilience, thereby consolidating the effectiveness of karst desertification control, enhancing GESs, and helping rural revitalization.

## 1. Introduction

Ecosystem services are the advantages that humans derive from ecosystems and are the bridge between ecosystems and human society [1,2]. Because of their unique above-ground and underground structures, karst areas are prone to ecological and environmental problems, such as soil erosion and vegetation degradation, due to the large fluctuation in their original site structure and topography. Among them, karst rocky desertification is an extreme manifestation of ecological degradation. Since 1989, China has carried out a great deal of work on issues related to rocky desertification in southwestern karst areas [3]. The construction of the Changzhu shelter belt has been successively implemented [4,5], including natural forest protection; forestry key points, such as returning farmland to forest (grass); a series of major national ecological projects, such as ecological projects, poverty alleviation and relocation in different places, rocky desertification comprehensive control projects, and pilot projects for comprehensive control of soil erosion on sloping farmland; and control of karst rocky desertification from different perspectives [3]. The aim is to develop ecological agriculture and animal husbandry in rocky desertification areas to increase residents’ income. Advancements have been made in the control of karst rocky desertification in southern China, and the control task is entering a new stage of improving ecosystem services [6]. After more than 30 years of comprehensive prevention and control of karst rocky desertification in southern China, remarkable achievements have been made. However, the structure of the grassland ecosystem in the control of rocky desertification is simple, the system function is incomplete, and the ecosystem sensitivity is relatively high. Ecological problems are still the shortcoming of ecological civilization construction and rural revitalization in karst rocky desertification areas. Therefore, enhancing the resilience, stability, and service capacity of regional ecosystems, consolidating the achievements of stone desertification management, and improving ecosystem services are important measures to improve people’s well-being. They also provide an important means to solve the ecological problems of stone desertification and an inevitable choice to guarantee the supply of ecological services and ecological security.

Vulnerability was first used in social science research and is now more commonly used in ecological research [7]. The definition of vulnerability has long been considered a “degree”, “capacity”, or “possibility” applied to a collection of concepts, including “sensitivity”, “response ability”, “exposure degree”, “adaptability”, “resilience”, and other elements. It was then applied to the concept of human–land coupling systems, which integrate the characteristics of nature, economy, society, humans and the environment, organizations, and institutions [8,9]. Resilience is mainly used in the field of mechanics to describe the ability of metals to recover after being deformed by external forces [10]. Holling first introduced the concept of resilience into the field of ecology to define the characteristics of the stable state of an ecosystem, which is the ability of an ecosystem to recover to a stable state after being disturbed [11]. Ecological resilience refers to the ability of an ecosystem to return to a stable state after a disturbance [11] and is used to measure an ecosystem’s ability to resist disturbance, a different measure of ecosystem vulnerability. The resilience of an ecosystem directly determines the supply of ecosystem services, constrains regional socioeconomic development, and affects human well-being. The anti-interference ability of ecosystems directly determines their stability and resilience, especially in fragile karst desertification areas. The vulnerability, resilience, structure, and stability of an ecosystem and ecosystem service supply capacity are system elements that are crucial to human well-being and constrain the sustainable development of human society.

In karst areas, the planting of grassland occurs mainly through the introduction of *Lolium perenne* L., *Pennisetum sinese Roxb*, and *Medicago sativa* L. or the sowing of *Trifolium repens* L. to form artificial grassland. Otherwise, it is based on replanting white clover, grazing to improve natural grassland, or the formation of rocky desertification management grassland ecosystems. The benefits of control over the years have shown that restoring and reforming natural grassland in karst areas can both maintain water and soil and improve karst stone desertification soil [12]. It has been confirmed that planting forage and ameliorating grassland are effective measures for karst desertification control. However, the internal mechanism of grassland ecosystem services for karst rocky desertification control have not yet been clarified, and they are not consistent with regional service demands. Therefore, there is an urgent requirement to improve the level of regional ecosystem services. However, there are very few studies on the ecological vulnerability, resilience, and enhancement of ecosystem services in karst rock desertification control. In particular, less research has been reported that closely integrates ecological vulnerability, ecological resilience, and ecosystem services. The grassland ecosystems of karst rocky desertification control are highly susceptible to ecosystem degradation due to their single structure and simple functions, as well as the highly sensitive background of rocky desertification—which restricts the enhancement of ecosystem service capacity, threatens regional ecological security, and limits economic development.

Although karst rocky desertification has been managed for more than 30 years, there are still many ecological and socioeconomic problems [13]. In the context of an ecological civilization construction and rural revitalization strategy, the way in which to scientifically promote karst desertification control and enhance the quality and stability of ecosystems has become an important issue for the harmonious coexistence of humans and nature in karst rocky desertification areas. Ecosystem quality and stability are closely related to ecosystem fragility and resilience, and ecosystem quality directly depends on ecosystem service capacity [14]. At present, the development of an appropriate method for regulating karst rocky desertification control ecosystems, reducing ecological vulnerability, enhancing ecological resilience, and improving ecosystem services is a key scientific issue that needs to be solved urgently.

In view of this, with the aim of enhancing the service function of grassland ecosystems in rocky desertification control, the progress and characteristics of grassland ecological vulnerability research are reviewed herein. Additionally, the intrinsic relationship between grassland ecosystem vulnerability, resilience, and services is explored in order to provide enlightenment on ideas to enhance the grassland ecosystem services of karst rocky desertification control, to consolidate the sustainability of rocky desertification control and regional ecological security barriers, to enhance the supply of grassland ecosystem services, and to promote the sustainable development of the regional ecological environment and social economy.

## 2. Methods

**Figure 1:**
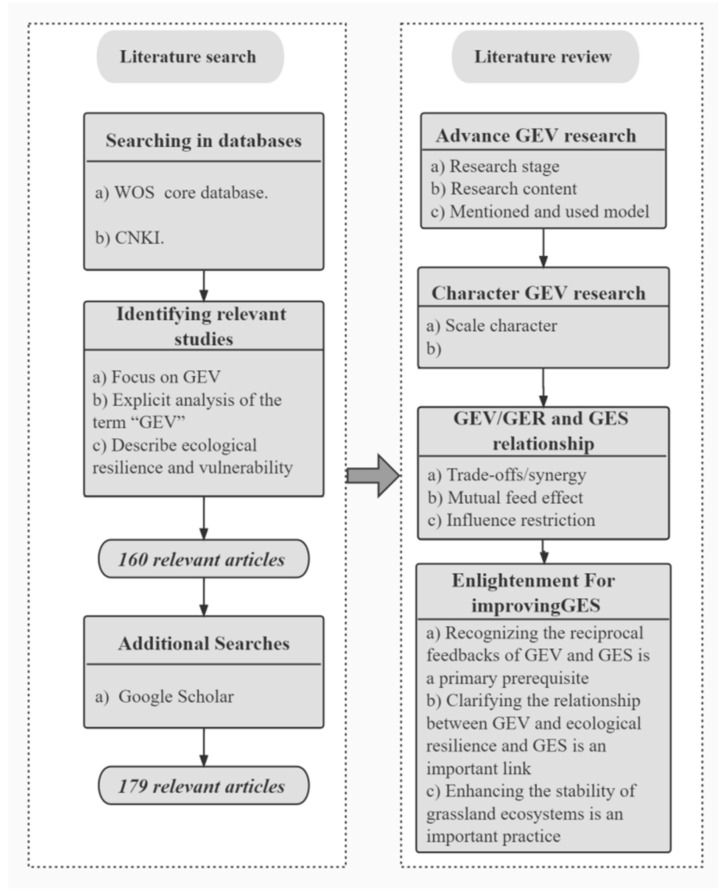
Flowchart showing the systematic literature search and literature review. “Trade-offs/synergy”: Trade-offs are situations in which the supply of some types of ecosystem services decreases as the use of other types of ecosystem services increases, while synergies are situations where two or more ecosystem services are enhanced simultaneously. “Mutual feed effect”: Mutual feedback effects, to a certain extent, are mutual causes and effects. “Influence restriction”: Certain restrictions or limitations on one another. GEV, grassland ecosystem vulnerability; GER, grassland ecological resilience; GES, grassland ecosystem service.

This review was performed in accordance with the PRISMA (Preferred Reporting Items for Systematic Reviews and Meta-Analyses) guidelines. This review was conducted based on a literature search and a systematic review, including quantitative statistics and qualitative content analysis (Figure 1). Systematic reviews have an advantage over traditional reviews and commentaries in that they cover studies by following an explicitly formulated procedure [15,16].

### 2.1. Literature Search

In this paper, to identify relevant studies, a search was conducted in the China National Knowledge Infrastructure (CNKI), which is the largest and most comprehensive Chinese literature database, and the Web of Science (WOS) core database for articles, publications, and conferences. In the Web of Science core database, the following syntax was used: TS = (grassland ecological vulnerability * or grassland ecosystem vulnerability * or grassland ecological fragility * or grassland ecosystem fragility * or steppe ecosystem vulnerability * or steppe ecological vulnerability * or steppe ecological fragility * or steppe ecosystem fragility * or prairie ecosystem vulnerability * or prairie ecological vulnerability * or prairie ecosystem fragility * or prairie ecological fragility) and (ecological resilience*) = English. A total of 500 unique articles were returned from the database. Meanwhile, in the CNKI database, “topic” was used as the search item, and “grassland” and “steppe” as the search words for the first search. Among the results, “ecosystem vulnerability” and “ecological resilience” were used as the search words for the second search. A total of 756 articles were returned from the database.

The acquired literature was screened at the title and abstract level and followed three criteria: ① focus on grassland ecosystem vulnerability; ② explicit analysis of the term grassland ecosystem vulnerability; or ③ related studies on grassland ecosystem resilience. During this process, 160 pertinent studies were retained. We also carried out additional searches in Google Scholar and identified 19 further articles closely related to GEV and GER. The final analysis was based on 179 articles. The top 10 contributors in number of Literature of the topic are Wang Ying (7), Bellocchi Gianni(5), Briske David D.(4), Guo Luo(3), Hou Xiang-Yang(3), Li Ping.(3), Li Rong(3), Shi Honghua(3), Bailey Derek W.(2) and Chen Jiquan.(2).

### 2.2. Literature Review

**Figure 2:**
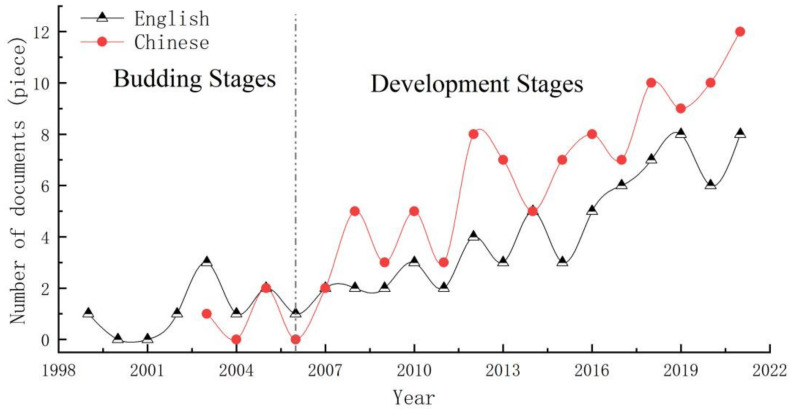
Grassland ecosystem vulnerability study stages: (1) statistics on the vulnerability and resilience of grassland ecosystems in each year; (2) based on the CNKI, WOS, and Google Scholar database retrieval and screening of the literature quantity and research content, the division of the research into stages (emergence and development stages).

We first reviewed advances in grassland ecosystem vulnerability research. According to the research themes that appeared in different years, the research phases were divided, which gradually increased with the passage of time, into two phases—budding and development (Figure 2)—and the two research phases were systematically discussed. We also condensed the scientific problems faced by the current grassland ecosystem vulnerability research. Then, we analyzed the characteristics of the selected grassland ecosystem vulnerability studies, investigating the relationship between grassland ecosystem vulnerability, ecological resilience, and ecosystem services based on articles related to ecological vulnerability, resilience, and services. Finally, we provide ideas for reference to enhance GESs for karst desertification control.

## 3. Advances in Grassland Ecosystem Vulnerability Research

Vulnerability is derived from the Latin word “vulnerare”, meaning “likely to be injured” [17], and is related to the concepts of risk, sensitivity, exposure, stability, and adaptive capacity [18,19]. Additionally, it is often referred to as the ability of an ecosystem to respond to stressors in a given time and space domain [20]. It first emerged in the field of natural hazards, where the likelihood of occurrence and the impact of hazards were used to identify and predict the risk areas of vulnerable groups [21]. As the field of research has expanded, the meaning of vulnerability has evolved from a collection of elements such as the “ability to cope with damage or disturbance, exposure, adaptive capacity, resilience, sensitivity” to the vulnerability of coupled human–earth systems that incorporate natural, socioeconomic, and other characteristics. In 1905, Clements mentioned the term “Ecotone” (ecological transition zones) in his book Research Methods in Ecology, linking vulnerability with the ecological environment, which attracted the attention of the ecological community [22]. The seventh SCOPE Conference confirmed the concept of Ecotone: The “interface” between two or more material, energy, structural, or functional systems in an ecosystem, and the “transition zone” that extends outward around this interface—“the spatial domain of the transition zone” [23]. Ecosystem vulnerability is used to measure the ecological exposure, sensitivity, and adaptive capacity exhibited by a stressed ecosystem. Exposure is the degree to which a system is exposed to a particular stressor; sensitivity is the degree to which an ecosystem responds to a stressor; and adaptive capacity is the potential of a system to adjust or respond to shocks [24]. 

### 3.1. Budding Stage

Research at the embryonic stage has focused on the exploration of the concept of ecosystem vulnerability, the analysis of vulnerability factors, and the qualitative evaluation of vulnerability. In terms of exploring the concept of ecosystem vulnerability, Metzger et al. argued that ecological vulnerability is a mapping of climate impacts to the adaptive capacity of a system in the context of climate change [25]. Williams and Kapustka argued that ecological vulnerability is the potential of an ecosystem to respond to temporal and spatial pressures and is a measure of an ecosystem’s ability to withstand pressures in time and space [26]. The above scholars considered the concept of ecosystem vulnerability mainly in terms of the structural function of the ecosystem itself and the natural environment and only described the inherent vulnerability of ecosystems, while the impact of human activities on ecosystems has rarely been taken into account. Ecological vulnerability is a characteristic of the relationship between humans and nature, reflecting the sensitivity of the natural environment [27], while Niu definition of ecological vulnerability zones takes into account the impact of human activities on ecosystems, making the concept of ecological vulnerability more complete [23]. Today, the concept of ecological vulnerability has evolved from a focus on sensitivity to damage or disturbance to a focus on a system’s ability to withstand, respond to, and regulate stimuli, as well as its ability to recover after being stimulated. The understanding of ecological vulnerability varies somewhat among researchers, but all encompass both the sensitivity of a system and its resilience after a disturbance [25].

In the context of global change, the fragile substrate of ecosystems and the interaction of anthropogenic disturbances lead to ecosystems exhibiting vulnerable properties. Therefore, ecological vulnerability factors can be divided into two categories: Natural environmental factors and socioeconomic factors. The natural environment is the foundation of the ecosystem and the cornerstone for maintaining the dynamic balance of the system, while human activities are the main drivers of ecosystem vulnerability. One study chose GPP to analyze the dynamic indicators of grassland degradation and used the residual trend method to assess the drivers of grassland degradation based on GPP, which showed that climate change is the main driver, and the spatial heterogeneity of human activities is significant [26]. In a study of ecological vulnerability factors in the Tutun River basin, Liu et al.concluded that the interaction of any two factors shows a non-linear reinforcing effect, and that the interactions of land use type ∩ elevation, land use type ∩ precipitation, and land use type ∩ temperature are significant controlling factors for ecological vulnerability [27]. That is, the vulnerability of ecosystems is the result of the interaction of natural and human factors. Although human activities are the main driver of ecological vulnerability, human and social factors are also major forces in reducing ecological vulnerability. In their study of the impact of conservation and restoration on reducing ecological vulnerability, Li et al. found that the role of natural factors has increased and that increased vegetation cover and economic development have contributed to reducing ecological vulnerability [28]. Ding et al. argued that anthropogenic factors have a greater impact on ecological vulnerability than natural factors, with landscape evenness and the degree of land resource use being the main factors influencing ecological vulnerability [29]. Fundamentally, vulnerability as an essential attribute of ecosystems is objective; although ecosystem vulnerability is caused by the interaction of both natural and human–social factors, human activities are only external drivers, and reasonable human activities can facilitate the realization of ecosystem services and can enhance human well-being—the two are opposites, that is, and human activities mutually constrain and influence one another.

Ecosystem vulnerability assessment is the exploration of the vulnerability characteristics of ecosystems and their environment, including the sources, status, drivers, and processes of vulnerability. Research on the empirical evaluation of ecological vulnerability has developed rapidly, and most studies on ecosystem vulnerability have taken typical ecologically fragile zones as the object of study [25]. The evaluation methods of ecological vulnerability at this stage are relatively simple, lack the support of scientific data systems, have low accuracy, and are qualitative in nature. For example, Ye et al. evaluated the degradation of grassland ecosystems based on GIS and found that rodent infestation, grazing intensity, and surface condition are the main causes of grassland degradation [30]. In his study of alpine grassland ecosystems in Qinghai, Cai found that over-grazing, over-cultivation, indiscriminate dredging, climate warming, and rodent, insect, and poisonous grass damage are the main causes of grassland ecosystem dysfunction [31].

An analysis of studies at the embryonic stage reveals that there is still room for a deeper investigation into the theoretical perception of the vulnerability of grassland ecosystems. In order to explore the vulnerability mechanisms of ecological grassland systems, scholars have moved from the description of vulnerability concepts to the exploration of ecological vulnerability research methods. Influenced by the trend of positivism and landscape ecology, the study of land use change and spatial differentiation structure based on landscape ecology has become the main focus of quantitative ecological vulnerability research [25]. This takes into account the gradual shift from qualitative description to quantitative analysis in studies. The quantitative assessment is a prerequisite and basis for understanding the ecological status of grasslands, formulating sustainable development plans, realizing ecosystem services, and enhancing human well-being [27,32]. However, studies on the vulnerability of grassland ecosystems in karst areas have still not been thoroughly investigated.

### 3.2. Development Stage 

As the research gradually deepens, in the development stage, it mainly focuses on the evaluation and prediction of grassland ecological vulnerability, optimizing the selection of evaluation indicators, exploring the mechanism of ecological vulnerability based on the context of climate change, and exploring the restoration and protection of ecosystems under the perspective of resilience. This reflects the deepening of the direction and content of research in the context of global change and sustainable development policies and the sustainability and long-lasting nature of human development, providing a basis for supporting decision making and optimizing ecosystem resilience and service enhancement in relevant regions.

Ecological vulnerability assessment is a primary prerequisite for effective ecosystem control and sustainable development [33] and has now evolved from qualitative research to quantitative measurement and prediction [34]. Ecological vulnerability assessments can be divided into three categories [33]: (1) risk assessments to understand the response of ecosystems to hazards [35]; (2) ecological vulnerability assessment approaches that combine natural threat factors with social factors [36,37]; and (3) integrated evaluation models that use theoretical frameworks, such as ecosystem services or resilience, and integrate these theories [18,38]. For instance, some scholars have quantitatively evaluated or predicted the vulnerability of ecosystems by combining the dominant factors in the vulnerability characterization of their study, intrinsic factors such as climate, soil, topography, and geology, and anthropogenic factors such as population, economic indicators, and spatial patterns [39]. Some researchers have used a combination of exposure, sensitivity, and resilience to evaluate the spatial pattern of climate change vulnerability in grasslands and to identify the drivers, showing that climate exposure controls the spatial pattern of vulnerability in alpine grasslands and that climate change sensitivity and resilience may also exacerbate or mitigate vulnerability in alpine grasslands [40]. Considering only the effects of climate change, a probabilistic-based assessment of the future risks to Mongolian grasslands suggests that climate change is the main cause of increased drought risk and vulnerability in grasslands [41]. In view of this, climate change is a predisposing factor for ecological vulnerability. On the contrary, Hao, constructed an evaluation index system based on the structure, biomass, climate, environmental factors, and socioeconomic conditions of grassland forage and used the AHP and GIS techniques to zone and analyze the causes, indicating that climatic conditions are the determining factor for the distribution of grassland types and the formation of grassland net primary productivity, and that under the influence of human activities, the overall condition of grassland ecology in Inner Mongolia is not optimistic, the degree of ecosystem vulnerability is high, and the ability to resist external disturbances is poor [42]. The above studies show that climate change has a significant impact on ecological vulnerability, and in future studies, focus needs to be on climate change, as a research context and inducing factor, to uncover its triggering mechanism in terms of ecosystem vulnerability and to explore its reciprocal feedback with ecological vulnerability.

The scientific selection of evaluation indicators is the first prerequisite for an accurate assessment of the vulnerability of grassland ecosystems. Evaluation indicators are a representation of the vulnerability factors of a system, and the dominant vulnerability factors of different systems are different; therefore, it is necessary to construct an indicator system applicable to different systems. There are three main types of ecological vulnerability evaluation indicators (Table 1): (1) a single type of vulnerability indicator system based on the geographical characteristics of the ecosystem and the dominant vulnerability factors in the context of regional environmental conditions [43]; (2) a comprehensive indicator system that integrates natural–social–economic factors and integrates the internal structure and function and the external disturbances of the ecosystem [44]; (3) in the context of global and climate change, indicator data provided to simulate and predict the vulnerability trends of ecosystems [45].

In terms of ecosystem fragility, researchers have carried out a great deal of research and have developed numerous research methods and models (Table 2 and Table 3)—for example, based on the SRP (sensitivity–resilience–pressure) model, which is a three-dimensional representation of ecosystem vulnerability, sensitivity, resilience, and stress. Liu Jialu et al. constructed evaluation indicators from natural and social factors and used RS, GIS technology, and principal component analysis to determine the weights of indicators to quantitatively assess the degree of ecological vulnerability in the Qilian Mountains Water Containment Area [46]. As the quantitative evaluation and prediction of ecological vulnerability continue to advance, scholars have gradually realized that it is necessary to evaluate both the level of vulnerability and the pattern and probability of occurrence of ecological vulnerability [34]. For instance, Rolinski et al. conducted a probabilistic risk assessment of the vulnerability of the carbon cycle of extreme weather in Europe based on an ecosystem perspective and assessed the vulnerability and risk of biomass loss in the event of drought [47]. Ecosystem vulnerability indicators are the basis for an accurate assessment and prediction of ecosystem vulnerability and risk, and global climate change is causing subtle changes in ecosystems and revealing specific properties that pose great challenges for the specification and harmonization of evaluation indicators.

In the context of global and climate change and rapid economic development, as well as the dynamics and regional heterogeneity of vulnerability factors, the study of ecosystem vulnerability drivers and evolutionary mechanisms has gradually become a hot topic of research [64]. In addition to analyzing the characteristics of ecosystem vulnerability, evaluation results, and spatial differentiation, researchers have also analyzed the drivers of vulnerability and their interactions and have explored the long-term evolution mechanism of regional ecosystem vulnerability [65]; have proposed early warning and prevention mechanisms for ecological vulnerability, combined with regional social development, from the perspectives of socioeconomic, resource and environmental, and ecological regulation; and have explored countermeasures and recommendations for ecosystem vulnerability. Studies have proposed countermeasures to explore the crises faced by ecosystems and established a vulnerability warning and prevention mechanism [38], as well as restored and rebuilt damaged ecosystems and highlighted optimization strategies based on the vulnerability status of ecosystems and the structural and functional characteristics of the system [66].

Resilience is a common characteristic of complex ecosystems, i.e., the ability of a system to respond to perturbations, internal failures, and environmental events to maintain its function by absorbing or reorganizing them [67], and is the same as elasticity—a measure of an ecosystem’s ability to recover from a disturbance [68], i.e., ecological resilience includes the self-organization, adaptive capacity, and absorptive capacity of the system [67]. That is, under a resilience perspective and external disturbances, grassland ecosystems are able to regulate themselves in a self-organizing, adaptive, and resilient manner, so that the ecosystem’s structure and function reach a new state of dynamic equilibrium. Learning the ecological resilience of grasslands is important for increasing their biological and habitat diversity, increasing their economic value, improving the function of their ecosystems, and enhancing their resistance to disturbance [69]. For example, microbial biomass, enzyme stoichiometry, and mass-specific enzyme activities have been used instead of the weighted average characteristics of microbial communities as a way to infer cascade effects between microbial resource use strategies and ecosystem resilience; to characterize ecosystem resilience by quantifying ecosystem processes and properties related to nitrogen cycling during pre-, mid-, and post-drought periods [70]; and thus, to provide scientific recommendations for regional ecological restoration.

In general, human–earth system coupling provides a perspective to mitigate the vulnerability of grassland ecosystems, enhance their adaptability and resistance to disturbance, and in turn, enhance their service functions and human well-being. In the case of grassland ecosystems under karst desertification control, the cascading patterns between grassland ecosystem structures, processes, services, and well-being should be grasped. Additionally, the impact of changes in GESs on karst environmental effects should be comprehensively considered, especially the current situation of grassland ecosystems under rock desertification control, their vulnerability and resilience, and the services they provide, while the evaluation and prognosis of typical grassland ecosystems under karst rocky desertification control should be assessed. It is also important to evaluate and predict the current situation and development trend of vulnerability of typical karst rock desert-managed grassland ecosystems, to analyze the mechanism of multiple contexts, to select evaluation indicators under multiple drivers, to predict and regulate vulnerability and prevention mechanisms, and to optimize grassland ecosystem resilience so as to synergize the relationship between resilience, vulnerability, and services and to enhance the function of GESs in karst desertification control. 

However, grassland ecosystem vulnerability depends on the type of grassland (e.g., meadow, true, or desert steppe) and even more so on the degradation status [71]. Tropical grasslands are highly resistant to systemic endogenous disturbances (fire, herbivores, etc.), but are sensitive to external disturbances (farming, mining, etc.) and are prone to ecological degradation [72]. Both desert and typical grasslands respond weakly to climate warming, but typical grasslands respond very strongly to watering, and both desert and typical grasslands effectively improve the soil carbon content and enhance soil respiration under watering conditions [73]. However, desert grasslands are more sensitive and vulnerable than typical grasslands if under the same conditions [74,75]. Unreasonable anthropogenic disturbance is the main factor leading to the loss of resilience and the ecological fragility of grassland ecosystems. Long-term and continuous human disturbances have led to a steady decline in the stability and diversity—and even a loss—of savanna ecosystems [76]. There are also differences in the vulnerability and resilience of natural versus artificial grasslands: natural grasslands are more resistant to disturbance, due to their rich species and complete system structure [77,78].

### 3.3. The Key Scientific Problems

① For the problem of the mechanism of the vulnerability of grassland ecosystems being unclear, through in-depth analysis of the ecosystem vulnerability factor and its mechanism and exploring the evolutionary law, the sustainable development of vulnerable ecosystems will be promoted, and the system service capacity will be improved. 

② Aimed at the problem of there being many indicators for evaluating the vulnerability of grassland ecosystems and a lack of unified standards, a set of universally applicable evaluation index systems has been constructed by comprehensively considering natural, socioeconomic, and ecological factors [49,79]. 

③ In view of the uncertainty of quantitative research on grassland ecosystem vulnerability, the analysis methods and models have been selected comprehensively through the study area, object characteristics, and scale to improve the accuracy of studies [80].

④ In view of the lack of theoretical and practical application of grassland ecosystem vulnerability research, according to the results of previous vulnerability research combined with regional characteristics, ecological restoration can be carried out, the combination of theory and practice can be promoted, and a virtuous circle of regional ecology can be championed [81].

⑤ Aimed at the problem of the grassland ecosystem vulnerability research framework being relatively simple, by constructing a diversified vulnerability research framework, the ecosystem vulnerability theory can be enriched. 

⑥ In view of the current situation of less research on grassland ecosystem resilience strategies, according to the research on grassland ecological weaknesses, research on ecological resilience mechanisms and resilience strategy improvement can be carried out, and the adaptive capacity and system service function of grasslands can be improved [82,83].

On the whole, scientific issues are the difficult problems faced by the current grassland ecosystem and even the entire body of ecosystem vulnerability research. In particular, the enrichment of ecosystem vulnerability research frameworks and the relative regularization of evaluation indicators are needed. Overcoming the above systematic problems is an important task for ecological researchers and is the key to promoting the dynamic balance of the ecological environment; it is of great significance to promote the study of ecosystem vulnerability.

## 4. Characterization of Grassland Ecosystem Vulnerability Studies

The retrieved literature was analyzed in terms of its research characteristics in relation to both its spatial scale and research content. The spatial scale can be divided into a large area scale based on the world and continents, and regional and watershed scales based on regions and administrative divisions (we provide a systematic discussion later). 

### 4.1. Large Area Scale

In the context of climate change, Christensen et al. used an ecosystem model to study the vulnerability of vegetation to climate change and grazing in a typical Asian grassland (Inner Mongolian grassland) [84]. Anjos et al. measured ecosystem resilience based on ecosystem climate ecotone using high-resolution remote sensing data and ecotone modeling techniques to calculate and spatialize three South American stable ecosystems (i.e., forests, savannas, and grasslands) and their resilience and found that intensifying climate change may lead to a loss of resilience in forest ecosystems, thereby significantly increasing the chances of key transition events to an alternative stable state with a lower vegetation cover density (e.g., savanna or grassland) [85]. As a result, grassland ecosystems are more resilient than forests under climate change. At the large area scale, research is biased toward evaluating or predicting ecosystem vulnerability using models based on the context of climate change, with the aim of analyzing the vulnerability of grassland ecosystems and predicting the ecological risks they may face to provide an objective basis for decision making to promote regional ecological security and resource use and control. For example, Zhang et al. constructed drought vulnerability curves between the aboveground net primary productivity (ANPP) and the drought intensity for forests and grasslands through global data collection, bias checking, and systematic integration, and they investigated the processes of sensitivity and adaptation to reveal vulnerability mechanisms [86]. 

### 4.2. Regional Scale

Research on the vulnerability of grassland ecosystems in different regions has also yielded valuable results. By exploring the resilience of South African savanna ecosystem functions under the influence of extreme climatic events (drought) and disasters (fire), Wilcox et al. provided systematic responses to the occurrence of extreme climatic events in savanna ecosystems [87]. The spatial pattern of grassland vulnerability to climate change has been elucidated by simultaneously integrating exposure, sensitivity, and resilience in the Tibetan Plateau region, thus identifying its driving forces. Spatial patterns of alpine grassland vulnerability have been shown to be constrained by climate exposure, and sensitivity and resilience to climate change may exacerbate or mitigate the degree of vulnerability, with grazing intensity being the most significant anthropogenic factor of ecological vulnerability on the Tibetan Plateau [40]. All of the above studies were based on climate change disturbances to explore the vulnerability and resilience of grassland ecosystems; in the context of global change trends, it is important for control, utilization, and ecological services to enhance ecological resources. Climatic factors are the natural driving force for ecological vulnerability, and human activities are the main driving force for the development of ecosystem vulnerability [88]. Studies have demonstrated that, under the dual action of human and natural factors, the grassland ecosystem environment is continuously degraded, which exposes the vulnerable attributes [89]. For example, grassland has a series of ecological problems such as fragmentation of the ecosystem landscape, changes in vegetation community diversity, and changes in structure and function [90]. In ecological risk assessment, a GIS-based grassland fire risk analysis and assessment method has been proposed from the multidisciplinary perspectives of climate, geography, and disasters, and a multidimensional grassland fire risk index has been developed to quantify grassland fire risk as a basis for regional resource allocation and planning [91].

### 4.3. Watershed Scale

At the watershed scale, Yao et al. used remote sensing and GIS techniques with a hierarchical analysis–principal component entropy method model to select indicators such as elevation, slope, and land use type to evaluate the ecological and environmental vulnerability of the middle and upper reaches of the Yalong River during 2000–2018. Additionally, they invoked the CA–Markov model to predict the ecological and environmental vulnerability of the region, thus revealing the dynamic change pattern and future development of ecological and environmental vulnerability in the region, and provided a theoretical basis for the formulation of ecological and environmental protection measures in the region [92]. Zhang et al. analyzed the changes in land use and cover in the upper reaches of Minjiang River from 2000 to 2010 and the changes in ecological sensitivity and fragility using NPP characterization, showing that the overall vulnerability/sensitivity of the upper watershed area is low, and the ecological vulnerability/ecological sensitivity is correlated with environmental factors (precipitation, drought index, etc.) [93]. This indicates that future studies of ecosystem vulnerability/sensitivity should pay more attention to the effects of climate change and human activities. For example, Chen et al. selected indicators reflecting regional characteristics (vegetation, hydrology, climate, topography, soil, human activities, etc.), used co-linear diagnostic analysis to construct an ecological vulnerability evaluation system for the Amu Darya River basin, and used an improved entropy weighting method integrating AHP and entropy weighting to determine indicator weights, which in turn quantified the ecological vulnerability and analyzed the spatial and temporal characteristics, with a deteriorating trend of the watershed ecological environment [94].

In summary, grassland ecosystem vulnerability studies at different spatial scales are based on different technical means and methods to evaluate the current state of fragility of grassland ecosystems, to predict ecological risks and strategies to cope with them, and to reduce ecological vulnerability. Meanwhile, climate change and human activities have been the inevitable background and causal factors considered in ecosystem vulnerability studies to enhance ecosystem stability and enhance ecosystem services for the purpose of enhancing human well-being.

## 5. Intrinsic Relationship between GES Enhancement and Grassland

### Ecosystem Vulnerability

GES refers to all of the benefits (including products, resources, and environments) that grassland biodiversity, ecosystem structure, and function provide to meet human needs for survival, livelihood, and well-being [95]. GES functions are closely related to grassland type, disturbance type and intensity, and species abundance [96], and it is clear that living organisms are the basis of ecosystem functions [97]. However, ecological reciprocity in grasslands promotes and protects biodiversity, which in turn forms a coupled system structure and ecological processes between organisms and the environment and regulates ecosystem functions through mechanisms that shape material, energy, and information flows—and in doing so, provides a variety of services to humans and other living species. Therefore, the special resource endowment of grasslands and their service functions provide inspiration for the control of karst rocky desertification, and also provide the basic conditions for regional ecological coordination and the development of grass and livestock industries.

Ecological vulnerability and resilience are different representations of an ecosystem’s response to disturbance [98], both of which can explain one another to some extent. That is, ecological resilience can be measured as a result of restoration or through attributes of uptake, adaptation, and responsiveness [99]. In other words, when grassland ecosystems are disturbed, ecological resilience can measure their sensitivity and environmental vulnerability, and vulnerability affects resilience only as a consequence of disturbance [100]. Both ecological vulnerability and ecosystem services are of interest to human well-being. In the context of global climate change, where the frequency of extreme weather events and the magnitude of the harm they cause are expected to increase, clarifying ecological resilience mechanisms is essential for predicting ecological vulnerability [87]. Ecosystem stability is correlated with ecosystem service functions, and ecological resilience, as a measure of ecosystem stability, is closely related to ecosystem services.

To some extent, ecosystem services can mitigate ecological vulnerability, but ecological vulnerability can also limit the performance of its services [101]. Therefore, improving the resilience of grassland ecosystems is significant for enhancing ecosystem services. The resilience of grassland ecosystems to disturbance is significantly related to their ability to provide services. With the intensified impact of human activities and climate change on grassland ecosystems, especially in karst desertification areas, the sensitivity of grassland ecosystems has significantly increased, while their anti-interference ability has significantly decreased. Therefore, the manner in which to improve the resilience and adaptive capacity of grassland ecosystems has become a top priority. An understanding of resilience is essential for developing the ability to predict the future structure, function, and services of ecosystems [102].

**Figure 3:**
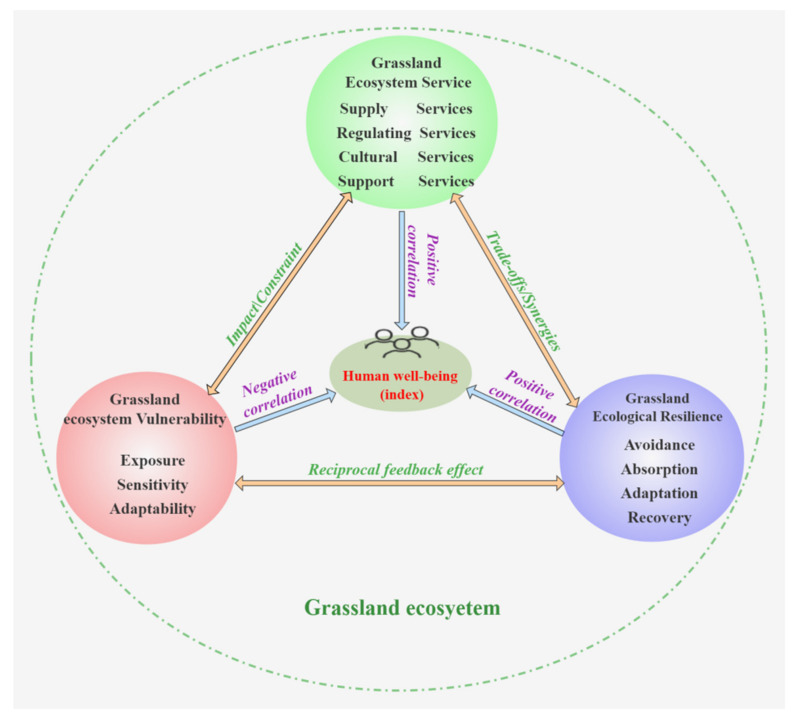
The relationship between grassland ecosystem vulnerability, resilience, and services.

In general, ecological vulnerability limits the realization of ecosystem service functions, while ecological resilience is the basic condition for the realization of ecosystem service functions; the realization of ecosystem service functions can effectively mitigate ecological vulnerability and enhance ecological resilience. Ecological vulnerability, resilience, and service functions are synergistic and constraining relationships within the ecosystem (Figure 3). Improving GESs is an important initiative to alleviate and enhance grassland ecological resilience. The already fragile substrate conditions and the influence of human activities in karst desertification areas make the fragility factors of grassland ecosystems more complex, the resilience mechanisms more difficult to explore, and the GES functions more difficult to realize. In karst areas, we should strengthen the investigation of the mechanisms of grassland ecosystem vulnerability and resilience, enhance the resilience of grassland ecosystems, alleviate ecological fragility, improve ecosystem services, promote ecological animal husbandry development, fundamentally improve the ecological environment, and consolidate the ecological and economic benefits of karst desertification control.

## 6. Research Advancement of Grassland Ecosystem Vulnerability and Ecological Resilience and Its Inspiration for Improving Grassland Ecosystem Services in Karst Desertification Control

### 6.1. Resilience and Vulnerability of Grassland Ecosystems in the Karst Desertification Control Area

Grasslands under karst desertification control have the ecological characteristics of karst areas. The vulnerability of karst ecosystems is mainly expressed via soil, hydrology, vegetation, and human environmental vulnerability [103]. Grasslands under karst desertification control are dominated by artificial grasslands and supplemented by improved grasslands, which constitute an important ecosystem in karst areas [104]. Grassland ecosystems have the problems of single species, a simple ecosystem structure, and weak ecosystem functions, so such ecosystems are very sensitive and easily degraded under exogenous disturbance, ultimately becoming very vulnerable [105]. Karst rocky desertification control grasslands are inherently vulnerable and weak in terms of system elasticity, especially in response to external pressures (such as farming and grazing). Vulnerability and ecological resilience of grassland ecosystems for karst stone desertification management comprise a relative set of concepts, with fragility being an expressive description of the state of grassland habitats and resilience being a description of the state of grassland ecological functions [66,106].

### 6.2. Research Advancement of Grassland Ecosystem Vulnerability and Ecological Resilience and Its Inspiration for Improving GESs in Karst Desertification Control

Grasslands are one of the most widely distributed ecosystem types on land [107] and play a significant role in developing livestock, maintaining biodiversity, preventing soil erosion, and maintaining ecosystem balance [108]. Grassland resources are an important foundation for building and advancing the sustainable and reliable development of livestock [109]. The unique geological characteristics of karst make the intrinsic characteristics of grassland ecosystems and the systematic structure–function architecture abound with vulnerability, as well as a lack of adaptation and sensitivity to external disturbances. The basal fragile environment of karst, coupled with irrational activities that exacerbate the fragile ecosystem, have led to further degradation of system functions, frequent geological hazards, and increasingly serious ecological and environmental problems [106]. Grass planting is one of the most effective ways to restore karst rocky desertification vegetation [110], which increases the grassland area and enriches karst grassland types in karst areas and provides the material basis for the development of karst livestock industry. Taking this into account, the ecological benefits of ecological protection can be realized, and the social benefits of regional economic development can be enhanced. In the context of the national rural revitalization strategy, the analysis based on the above-mentioned content has the following main points of insight.

#### 6.2.1. Adequate Understanding of the Reciprocal Feedback of Ecosystem Vulnerability and Functions Is the Primary Prerequisite for Enhancing GESs

Karst areas have achieved remarkable results in ecological restoration, such as karst rocky desertification control and revegetation, but there is an urgent need for effective methods to enhance ecosystem services [2]. Based on natural and socioeconomic factors, such as karst landscape features and land use changes, studies have been conducted on ecosystem service enhancement and value assessment in karst areas. To a certain extent, vulnerability can reduce its service functions, and the function of grassland ecological services can also regulate grassland ecological vulnerability. The vulnerability is determined by both internal (system structure, climate, etc.) and external (human activities) elements of the system, where human activities play a dominant role. Meanwhile, human activities affect the structure and function of the ecosystem, the supply and delivery of ecosystem services, etc. [111]. Therefore, in terms of the impact of human activities on ecosystems, which cause differences in ecosystem resilience, service supply is the main factor of trade-off/synergy between ecosystem vulnerability, resilience, and service functions in karst areas. Therefore, the reciprocal feeding mechanism of grassland ecological vulnerability, resilience, and ecosystem services is reflected in several aspects: ① grassland ecological resilience provides different quantitative representations of ecosystem disturbance; ② there is a degree of reciprocal effects between GESs and ecological vulnerability; ③ there is a non-linear coupling mechanism between grassland ecological resilience and ecosystem services; ④ in grassland ecosystems, ecological resilience and ecosystem services provide synergistic, constraining, and reciprocal feedback.

#### 6.2.2. Clarifying the Relationship between Ecological Vulnerability and Resilience Is an Important Part of Enhancing the Ecological Service Functions of Grasslands

An analysis of studies related to ecosystem vulnerability, resilience, and ecosystem services showed that the research objectives, objects, and models focused on the assessment and trade-offs of ecosystem states and values. For ecosystems, vulnerability may be more than just a balanced measure of structure and functions; resilience may also be more than just the average elasticity of the community [87]. Thus, both grassland ecological resilience and ecological vulnerability are related to the degree of disturbance to grasslands and are measures of different dimensions of disturbance to grassland ecosystems. Ecological vulnerability is a characterization of the sensitivity and exposure of grassland ecosystems and is concerned with changes in ecosystem state, while ecological resilience is a measure of system resilience (recovery) and is concerned with the sustainability of ecosystem dynamics [24]. Therefore, the ecological vulnerability of grasslands is consistent with the ecological resilience. In China, karst rocky desertification areas are mostly inhabited by ethnic minorities and are relatively impoverished areas. GESs are usually considered as the functions that grasslands can provide, such as production and supply, climate regulation, water conservation, soil and water conservation, and habitat and custom transmission. The production of grasslands is important for ecosystem services, such as carbon sinks, but changes in the biodiversity (grass species richness) of grasslands affect the quality and quantity of forage [87]; once the ecology of grasslands is degraded, it will also affect animal habitats, the local climate, and the regional cultural heritage. Therefore, combining the characteristics of karst rocky desertification control measures and effectiveness, the vulnerability and resilience of grassland ecosystems at different spatial and temporal scales are considered, and multi-scale and multi-type service flows are constructed; the trade-off/synergy relationships among the service flows and their scale dependence and spatial differences are clarified; the natural and socioeconomic factors affecting the spatial and temporal distribution and selection preferences of GESs are revealed—thus highlighting the beneficial effects and important link to enhance the service functions of grassland ecosystems.

#### 6.2.3. Reducing Ecological Vulnerability and Increasing Ecological Resilience Are Important Practices for Enhancing GESs

The sustainability of GESs requires understanding the threshold [112] and ecological threshold of grassland ecological resilience [113]. Even though ecological resilience is a key factor for ecological stability and ecological service function realization, enhancing GES functions depends on improvement of the service decision support capacity and the scientific understanding of the synergistic relationships among service flows and clusters, which are needed for ecosystem dynamic equilibrium maintenance and are necessary for mitigating grassland ecological vulnerability and enhancing ecological resilience. Inevitably, there are non-linear relationships, characteristic mechanisms, and spatial and temporal patterns between service streams and clusters in grassland ecosystems, and the application of the concept of sustainable development to clarify the trade-offs/synergistic relationships of service streams is beneficial to the long-term development of regional ecology. Contemporary research on ecosystem service flow interactions has made some progress, but it has not yet reached the level of decision-making applications in terms of their interaction characteristics, mechanisms, and modeling [114,115,116]. Therefore, in the study of GESs in karst areas, we can strengthen the assessment of negative products of GESs, enhance the linkage trajectories between service flows, optimize the ecological compensation, integrate GESs with ecological processes of karst desertification control, improve the relevant evaluation indicators and assessment methods, and promote the continuous deepening of GES research so as to achieve karst desertification control, rural revitalization, and sustainable development, as well as providing a pathway to enhance GESs.

## 7. Conclusions

The primary aim of this research was to analyze the ecosystem vulnerability and ecological resilience of grasslands. We reviewed the advanced and studied characteristics of grassland ecosystem vulnerability research and clarified the relationship between grassland ecosystem vulnerability, ecological resilience, and ecosystem services (Figure 3). We also analyzed the relationship between vulnerability and resilience for the karst desertification control of grassland ecosystems. Moreover, combined with a national ecological civilization construction and rural revitalization strategy, we provided three pointers for improving grassland ecosystem services for karst desertification control: ① the adequate understanding of the reciprocal feedback of ecosystem vulnerability and functions is the primary prerequisite for enhancing GESs; ② clarifying the relationship between ecological vulnerability and resilience is an important part of enhancing the ecological service functions of grasslands; and ③ reducing ecological vulnerability and increasing ecological resilience are important practices for enhancing GESs.

## Figures and Tables

**Table 1:** Types of ecological vulnerability assessment index systems.

Types	Characteristic
Single index system	A strong regional specificity and ability to accurately identify the key indicators of vulnerability; however, the structure is simple and non-comprehensive.
Comprehensive type	The scope is broad, comprehensive, and integrated; however, it contains a wide range of indicators and is less actionable due to the limitation of data availability.
Simulation prediction type	A system of indicators is established through data of change factors to simulate the ecological vulnerability of future systems; the method is mainly applied to the simulation of ecological sensitivity and the potential impacts.

**Table 2:** Ecological vulnerability assessment methods.

Research Direction	Evaluation Method	Ideas	Pros and Cons	Literature Source
Ecological vulnerability assessment	Fuzzy Evaluation Method	Determine the index system and weight, calculate the membership degree of each factor to each evaluation index, analyze the resulting vector, and then evaluate the vulnerability level of each region and rank it.	Pros: The calculation method is simple and easy to implement. Cons: Not sensitive enough to reflect the vulnerability of indicators.	[48]
	Ecological Vulnerability Index Evaluation Method	Determine the indicators, weight, and ecological thresholds, calculate the ecological vulnerability index EFI based on numerical standardization according to the formula, and divide the vulnerability level.	Pros: Closely integrates vulnerability assessment and environmental quality. Cons: The results are relative.	[49]
	Comprehensive Evaluation Method	Included three parts: status evaluation, trend evaluation, and stability evaluation.	Pros: It is relatively comprehensive and macroscopic, and the evaluation results are highly comprehensive, logical, and systematic. Cons: Complex, involves many contents, and difficult to apply on a large scale.	[50]
	Grey Relational Analysis	Calculate the relative weight of each factor, calculate the relative vulnerability of the region according to the formula, and analyze the time series of the factors to obtain the trend of the regional vulnerability.	Pros: It is possible to compare the vulnerability of adjacent ecosystems and the development and change of its own vulnerability. Cons: The calculation process is complicated.	[51]
	Landscape Ecology Model	Mainly through the calculation of landscape index (landscape dominance, landscape dominance index, landscape separation index, landscape fragmentation index, etc.), combined with the land use index or integration into the ecologically fragile area sensitivity index and other spatial heterogeneous changes to the research object; the characteristics and ecological environment effects are analyzed.	Pros: Simple and easy to understand, not very strict on the number of time samples, and able to reflect the ecological process. Cons: It is different from the real landscape process, the application scope is narrow, the process is too simplified, and the dynamic mechanism of the landscape is ignored.	[52]
Ecological vulnerability prediction method	Ecological Footprint	Calculate the ecological footprint and ecological carrying capacity index of the study area and compare the ecological footprint to the ecological carrying capacity index.	Pros: It can be combined with the stepwise regression method to evaluate the ecological vulnerability status of the study area. Cons: The calculation process is relatively complicated, and it is a static analysis method that can only evaluate the development status of the region.	[53]
	Scenario Analysis	By simulating the impact of climate change on the main functions of various systems, the vulnerability pattern under different climatic conditions can be studied.	Pros: A more accurate simulation result can be given for situations that will not change much in the future. Cons: There is greater uncertainty.	[54]

**Table 3:** Ecological vulnerability evaluation model.

Evaluation Model	Model Selection Indicator System Implications	Vulnerability Mechanisms	Literature Sources
Pressure–State–Response (PSR) model	Pressure indicators reflect the load on the system caused by human activities, state indicators are the result of the long-term action of various factors in the system, and response indicators are generally the countermeasures and measures taken by humans in the face of ecological problems.	Taking the environment, society, economy, and human activities as the starting point, it focuses on social and economic development while reflecting the state of development of the ecological environment and the positive and negative impacts of human activities.	[55,56]
Sensitive–Recovery–Pressure (SRP) model	Ecological sensitivity characterizes the possibility of causing ecological problems when an ecosystem is affected by external influences; ecological resilience is the ability of an ecosystem to self-regulate and repair when it is damaged; ecological stress refers to factors that interfere with the stability of an ecosystem.	An ecological evaluation index system model that integrates natural, human, and ecosystem internal change factors to comprehensively evaluate the ecological vulnerability of a specific region.	[57,58]
Vulnerability–Scoping–Diagram (VSD) model	Exposure is the proximity of the system to the hazard; sensitivity is the damage to the system under stress; resilience is the state of the system under stress and the ability of the system to recover after damage.	Starting from the connotation of vulnerability, analyze the ability of the system to resist interference, the ability to self-regulate and recover, and the probability of the system being exposed to dangerous interference.	[59,60]
Sensitive–Elastic–Pressure (SEP) model	Ecological sensitivity is the sensitivity of the ecological environment to various disturbances, reflecting the ability to resist disturbances; ecological resilience is the property of the ecological environment to self-regulate and self-restore when internal and external disturbances or pressures do not exceed its elastic limit; ecological pressure is the pressure on the ecological environment brought by human survival needs and socioeconomic activities.	Starting from the connotation of vulnerability, environment, ecological structure, society, economy, and human activities, it reflects the development status of the ecological environment and the current vulnerability of the ecological environment under human activities.	[61,62]
Ecosystem Structure–Function–Habitat Index System	The ecosystem structure includes indicators such as vegetation index and leaf area index; ecosystem functions include indicators such as total primary productivity and net primary productivity; the ecosystem habitat includes climate, topography, and environmental factors.	Structure, function, and habitat are used as indicator sets to reflect the sensitivity of ecosystems to external disturbances and their own adaptive capacity; to a certain extent, they reflect the status of natural disturbances and human activities.	[48]
Causes and Results	Including the causal indicators of the natural environment (climate, precipitation, etc.) and the performance results of the regional system (per capita GDP, population density, etc.).	From the connotation of vulnerability, it pays attention to the influence of the internal and external factors of the system, and to the natural conditions and the condition of human interference.	[63]

## Data Availability

Not applicable.

## References

[1] Turner W.R., Brandon K., Brooks T.M., Costanza R., Da Fonseca G.A.B., Portela R. (2007). Global Conservation of Biodiversity and Ecosystem Services. BioScience.

[2] Xiong K.N., Xiao J., Zhu D.Y. (2022). Research progress of agroforestry ecosystem services and its implications forindustrial revitalization in karst regions. Acta Ecol. Sin.

[3] Ke Q., Zhang K., Wang A., He J., Zhang S. (2021). Database Construction and Mulyi-scale Intergrated Arrangement of Eco-technology for Combating Karst Rocky Desertification. J. Bull. Soil Water Conserv..

[4] Tan Q., Chen C., Zeng X., He J. (2018). Review and Prospect of Protection Forest System Construction in the Yangtze River Basin in the Past 30 Years. J. Sci. Soil Water Conserv..

[5] Fu J. (1993). Talking about the Construction of Shelter Forest System Engineering in the Pearl River Basin. J. For. Resour. Manag..

[6] Gao J., Xiong K. (2015). Ecosystem service value responses to ecological control in Karst region—A case study of Huajiang Gorge Demonstration Area of Rocky Desertification Control in Guizhou. J. Chin. J. Eco-Agric..

[7] De Lange H., Sala S., Vighi M., Faber J. (2010). Ecological vulnerability in risk assessment—A review and perspectives. Sci. Total Environ..

[8] Birkmann J. (2006). Measuring Vulnerability to Hazards of National Origin.

[9] Deng Y., Chen W., Luo S., Meng Q., Shi W., Li L. (2020). Research of Concept of “Landscape Vulnerability of Tourist Caves” and its Evaluation. J. J. Southwest Univ..

[10] Shao Y., Xu J. (2015). Understanding Urban Resilience: A Conceptual Analysis Based on Integrated International Literature Review. J. Urban Plan. Int..

[11] Holling C.S. (1973). Resilience and Stability of Ecological Systems. Annu. Rev. Ecol. Syst..

[12] Chi Y., Xiong K., Zhang Y., Dong Y., Liu C., Xu L. (2015). The beneficial results, problems and suggestions of grass-planting and livestock-raising to bring rocky desertification under control in the Karst areas of southwest China. J. Eilongjiang Anim. Sci. Vet. Med..

[13] He X., Wang L., Ke B., Yue Y., Wang K., Cao J., Xiong K. (2019). Progress on Ecological Conservation and Restoration for China Karst. J. Acta Ecol. Sin..

[14] Carpenter S.R., Brock W.A., Folke C., van Nes E.H., Scheffer M. (2015). Allowing variance may enlarge the safe operating space for exploited ecosystems. Proc. Natl. Acad. Sci. USA.

[15] Khan K.S., Kunz R., Kleijnen J., Antes G. (2003). Five Steps to Conducting a Systematic Review. J. R. Soc. Med..

[16] Vukomanovic J., Steelman T. (2019). A Systematic Review of Relationships Between Mountain Wildfire and Ecosystem Services. Landsc. Ecol..

[17] Birkmann J., Kienberger S., Alexander D.E. (2014). Introduction Vulnerability: A key Determinant of Risk and Its Importance for Risk Management and Sustainability. Assessment of Vulnerability to Natural Hazards.

[18] Füssel H.-M. (2007). Vulnerability: A generally applicable conceptual framework for climate change research. Glob. Environ. Chang..

[19] Darabi H., Hamedi R., Ehsani A., Kafi M. (2018). Rapid Vulnerability Assessment of Lavizan Urban Forest Park. Pollution.

[20] Beroya-Eitner M.A. (2016). Ecological vulnerability indicators. Ecol. Indic..

[21] Feng Q., Liu D. (2016). Research progress on vulnerability assessment of natural disasters in China. Adv. China Public Secur..

[22] MacMillan C. (1905). Research Methods in Ecology. By Frederic E. Clements, Ph.D. Lincoln, Nebraska, The University Publishing Company. 1905. Pp. xvii + 334. Science.

[23] Niu W. (1989). The discriminatory index with regard to the weakness, overlapness, and breadth of ecotone. J. Acta Ecol. Sin..

[24] Mumby P., Chollett I., Bozec Y.-M., Wolff N. (2014). Ecological resilience, robustness and vulnerability: How do these concepts benefit ecosystem management?. Curr. Opin. Environ. Sustain..

[25] Qu Z., Sheng T., Xu S., Liu Y., Han G. (2020). Review of ecological vulnerability evalution. J. Grassl. Prataculture.

[26] Yan H., Xue Z., Niu Z. (2021). Ecological restoration policy should pay more attention to the high productivity grasslands. Ecol. Indic..

[27] Liu Q., Shi T. (2019). Spatiotemporal Differentiation and the Factors of Ecological Vulnerability in the Toutun River Basin Based on Remote Sensing Data. Sustainability.

[28] Li Q., Shi X., Wu Q. (2020). Effects of protection and restoration on reducing ecological vulnerability. Sci. Total Environ..

[29] Ding Q., Shi X., Zhuang D., Wang Y. (2018). Temporal and Spatial Distributions of Ecological Vulnerability under the Influence of Natural and Anthropogenic Factors in an Eco-Province under Construction in China. Sustainability.

[30] Ye Y., Yang Z., Di B., Tang D., Wang C., Tang J. (2003). On grassland resource and the suatainable development of animal hus-bandry in Dingjie County. Tibet J. Mt. Sci..

[31] Cai D. (2006). Evaluation of Qinghai alpine grassland ecosystem, causes of functional disorders and management countermeasures. J. Pratacultural Sci..

[32] Ye X., Zhou H., Zhao X., Wen J., Chen Z., Duan J. (2011). Review on grassland ecosystem health. J. Pratacultural Sci..

[33] Darabi H., Farsani S.I., Behbahani H.I. (2019). Evaluation of Ecological Vulnerability in Chelgard Mountainous Landscape. Pollution.

[34] Yajun W., Lifang Z. (2020). Research Framework for Ecosystem Vulnerability: Measurement, Prediction, and Risk Assessment. J. Resour. Ecol..

[35] Papadopoulos G. (2016). Hazard, Vulnerability, and Risk Assessment Tsunamis in the European Mediterranean Region.

[36] Berrouet L., Machado J., Villegas-Palacio C. (2018). Vulnerability of socio-ecological systems: A conceptual Framework. Ecol. Indic..

[37] Maikhuri R.K., Nautiyal A., Jha N.K., Rawat L.S., Maletha A., Phondani P.C., Bahuguna Y.M., Bhatt G.C. (2017). Socio-ecological vulnerability: Assessment and coping strategy to environmental disaster in Kedarnath valley, Uttarakhand, Indian Himalayan Region. Int. J. Disaster Risk Reduct..

[38] Beier C.M., Patterson T.M., Chapin F.S. (2008). Ecosystem Services and Emergent Vulnerability in Managed Ecosystems: A Geospatial Decision-Support Tool. Ecosystems.

[39] Costanza R., de Groot R., Sutton P., van der Ploeg S., Anderson S.J., Kubiszewski I., Farber S., Turner R.K. (2014). Changes in the global value of ecosystem services. Glob. Environ. Chang..

[40] Li M., Zhang X., He Y., Niu B., Wu J. (2020). Assessment of the vulnerability of alpine grasslands on the Qinghai-Tibetan Plateau. PeerJ.

[41] Nandintsetseg B., Boldgiv B., Chang J., Ciais P., Davaanyam E., Batbold A., Bat-Oyun T., Stenseth N.C. (2021). Risk and vulnerability of Mongolian grasslands under climate change. Environ. Res. Lett..

[42] Hao R. (2015). Evaluation of the Impact of Climate Change on the Ecological Vulnerability of Main Grassland Areas in Inner Mongolia. The 32nd Annual Meeting of the Chinese Meteorological Society S6 Addressing Climate Change, Low-Carbon Development and Ecological Civilization Construction. https://cpfd.cnki.com.cn/Article/CPFDTOTAL-ZGQX201510006048.htm.

[43] Yun X., Hou X., Liu G., Yin Y. (2012). Research progress of vulnerability assessment on grassland ecosystem under climate change. J. Agric. Sci. Technol..

[44] Li B., Su F., Yang Z., Han Z., Peng F. (2018). Vulnerability-based analysis of the spatial-temporal dynamic patterns of the human-sea territorial system of the Bohai-rim region, China. J. Acta Ecol. Sin..

[45] Yu L., Cao M., Li K. (2005). An overview of assessment of ecosystem vulnerability to climate change. J. Prog. Geogr..

[46] Liu J., Zhao J., Shen S., Zhao Y. (2020). Ecological vulnerability assessment of Qilian Mountains region based on SRP conceptual model. J. Arid. Land Geogr..

[47] Rolinski S., Rammig A., Walz A., von Bloh W., van Oijen M., Thonicke K. (2015). A probabilistic risk assessment for the vulnerability of the European carbon cycle to weather extremes: The ecosystem perspective. Biogeosciences.

[48] Liu H., Wang N., Xie J., Zhu J. (2014). Assessment of ecological vulnerability based on Fuzzy Comprehensive evaluation in Weihe River basin. J. Shenyang Agric. Univ..

[49] Guo B., Zang W., Luo W. (2020). Spatial-temporal shifts of ecological vulnerability of Karst Mountain ecosystem-impacts of global change and anthropogenic interference. Sci. Total Environ..

[50] Guo J., Huang Y. (2016). Assessment of ecosystem vulnerability in Pingtan County based on AHP and Fuzzy Comprehensive eval-uation. J. Prot. For. Sci. Technol..

[51] Luo D., Zhang H. (2018). Grey incidence analysis method for regional drought vulnerability. J. North China Univ. Water Resour. Electr. Power.

[52] He J., You W., Hong W., Wu L., Zhan S., You H. (2018). Research advances in landscape ecology modeling in the latest 10 years. J. Southwest For. Univ..

[53] Su W., Li W., Zhu Y., Cai D., Yu C., Xu J., Wei W. (2019). Evaluation of sustainable development in Qinghai based on energy ecological footprint model. Pratacultural Sci..

[54] Yu L., Cao M., Tao B., Li K., Dong W., Liu H., Liu C. (2008). Quantitative assessment of the vulnerability of terrestrial ecosystem of China to climate change based on potential. Chin. J. Plant Ecol..

[55] Sun R., Yao P., Wang W., Yue B., Liu G. (2017). Assessment of Wetland Ecosystem Health in the Yangtze and Amazon River Basins. ISPRS Int. J. Geo-Inf..

[56] Zhang X., Wang L., Fu X., Li H., Xu C. (2017). Ecological vulnerability assessment based on PSSR in Yellow River Delta. J. Clean. Prod..

[57] Yao K., Yu L., Liu G., Liu H. (2017). Evaluation of ecological vulnerability in Sichuan province based on SRP model. Comput. Tech. Geophys. Geochem. Explor..

[58] Chen X., Li X., Eladawy A., Yu T., Sha J. (2021). A multi-dimensional vulnerability assessment of Pingtan Island (China) and Nile Delta (Egypt) using ecological Sensitivity-Resilience-Pressure (SRP) model. Hum. Ecol. Risk Assessment: Int. J..

[59] Polsky C., Neff R., Yarnal B. (2007). Building comparable global change vulnerability assessments: The vulnerability scoping diagram. Glob. Environ. Chang..

[60] Li Y., Fan Q., Wang X., Xi J., Wang S., Yang J. (2015). Spatial and temporal differentiation of ecological vulnerability under the frequency of natural hazard on SRP model: A case study in Chaoyang County. Sci. Geogr. Sin..

[61] Qiao Q., Gao J., Wang W., Tian M., Lv S. (2008). Method and application of ecological frangibility assessment. Res. Environ. Sci..

[62] Huang Y., Su J., Lv F. (2017). Evaluation method of land resources carrying capacity based on SEP model: A case of Minhang district in Shanghai. China Popul. Resour. Environ..

[63] Zhang X., Yu W., Cai H., Guo X. (2018). Review of the evaluation methods of regional eco-environmental vulnerability. Acta Ecol. Sin..

[64] Yang F., Ma C., Fang H. (2019). Research progress on vulnerability: From theoretical research to comprehensive practice. Acta Ecol. Sin..

[65] Füssel H.-M., Klein R.J. (2006). Climate Change Vulnerability Assessments: An Evolution of Conceptual Thinking. Clim. Chang..

[66] Hou W., Gao J., Peng T., Wu S., Dai E. (2016). Review of ecosystem vulnerability studies in the karst region of Southwest China based on a structure-function-habitat framework. Prog. Geogr..

[67] Fraccascia L., Giannoccaro I., Albino V. (2018). Resilience of Complex Systems: State of the Art and Directions for Future Research. Complexity.

[68] Bastiaansen R., Doelman A., Eppinga M.B., Rietkerk M. (2020). The effect of climate change on the resilience of ecosystems with adaptive spatial pattern formation. Ecol. Lett..

[69] Song M., Liu L., Chen J., Zhang X. (2018). Biology, multi-function and optimized management in grasslang ecosystem. Ecol. Environ. Sci..

[70] Piton G., Legay N., Arnoldi C., Lavorel S., Clément J.-C., Foulquier A. (2019). Using proxies of microbial community-weighted means traits to explain the cascading effect of management intensity, soil and plant traits on ecosystem resilience in mountain grasslands. J. Ecol..

[71] Valkó O., Zmihorski M., Biurrun I., Loos J., Labadessa R., Venn S. (2016). Ecology and Conservation of Steppes and Semi-Natural Grasslands. Hacquetia.

[72] Buisson E., Le Stradic S., Silveira F.A.O., Durigan G., Overbeck G.E., Fidelis A., Fernandes G.W., Bond W.J., Hermann J.-M., Mahy G. (2018). Resilience and restoration of tropical and subtropical grasslands, savannas, and grassy woodlands. Biol. Rev..

[73] Xu Z., Hou Y., Zhang L., Liu T., Zhou G. (2016). Ecosystem responses to warming and watering in typical and desert steppes. Sci. Rep..

[74] Liu X., Zhu Z., Yu M., Liu X. (2021). Drought-induced productivity and economic losses in grasslands from Inner Mongolia vary across vegetation types. Reg. Environ. Chang..

[75] Schermer M., Darnhofer I., Daugstad K., Gabillet M., Lavorel S., Steinbacher M. (2016). Institutional impacts on the resilience of mountain grasslands: An analysis based on three European case studies. Land Use Policy.

[76] MacDougall A.S., McCann K.S., Gellner G., Turkington R. (2013). Diversity loss with persistent human disturbance increases vulnerability to ecosystem collapse. Nature.

[77] Wang S.-Y., Liu J.-S., Yang C.-J. (2008). Eco-Environmental Vulnerability Evaluation in the Yellow River Basin, China. Pedosphere.

[78] Eek L., Zobel K. (2001). Structure and diversity of a species-rich grassland community, treated with additional illumination, fertilization and mowing. Ecography.

[79] Liu T.Y., Ye N.H., Wang X.Y., Das D.B., Tan Y.X., You X.K., Long M.X., Hu T.M., Dai L., Zhang J.H. (2021). Drought stress and plant ecotype drive microbiome recruitment in switchgrass rhizosheath. J. Integr. Plant Biol..

[80] Weißhuhn P., Müller F., Wiggering H. (2018). Ecosystem Vulnerability Review: Proposal of an Interdisciplinary Ecosystem Assessment Approach. Environ. Manag..

[81] Liang X.Y. (2018). Study on the Driving Mechanism of Macroscopical Land Use Function Change in Ecologically Fragile Area.

[82] Yan S.Y., Tang J. (2020). Progress on the Theory and Practice of Resilience City. J. Hum. Settl. West China.

[83] Sung C.-H., Liaw S.-C. (2021). Using Spatial Pattern Analysis to Explore the Relationship between Vulnerability and Resilience to Natural Hazards. Int. J. Environ. Res. Public Health.

[84] Christensen L., Coughenour M.B., Ellis J.E., Chen Z.Z. (2004). Vulnerability of the Asian Typical Steppe to Grazing and Climate Change. Clim. Chang..

[85] Anjos L.J.S., De Toledo P.M. (2018). Measuring resilience and assessing vulnerability of terrestrial ecosystems to climate change in South America. PLoS ONE.

[86] Zhang L., Gao J., Tang Z., Jiao K. (2021). Quantifying the ecosystem vulnerability to drought based on data integration and processes coupling. Agric. For. Meteorol..

[87] Wilcox K.R., Koerner S.E., Hoover D.L., Borkenhagen A.K., Burkepile D.E., Collins S.L., Hoffman A.M., Kirkman K.P., Knapp A.K., Strydom T. (2020). Rapid recovery of ecosystem function following extreme drought in a South African savanna grassland. Ecology.

[88] Liu X.-P., Zhang J.-Q., Tong Z.-J., Bao Y. (2012). GIS-based multi-dimensional risk assessment of the grassland fire in northern China. Nat. Hazards.

[89] Yao K., Zhang C., He L., Li Y., Li X. (2020). Dynamic evaluation and prediction of ecological environment vulnerability in the middle-upper reaches of the Yalong River. Remote Sens. Nat. Resour..

[90] Zhang J., Sun J., Ma B., Du W. (2017). Assessing the ecological vulnerability of the upper reaches of the Minjiang River. PLoS ONE.

[91] Chen T., Bao A., Guo H., Zheng G., Yuan Y., Yu T. (2019). Ecological vulnerability assessment for a transboundary basin in Central Asia and its spatiotemporal charcteristics analysis: Taking Amu Darya River Basin as example. J. Nat. Resour..

[92] Xie G., Lu C., Xiao Y., Zheng D. (2003). The economic evaluation of grasslang ecosystem service in Qinghai-Tibet Plateau. Mt. Res..

[93] Wang J., Chen Z.H. (2010). Modeling dynamic assessment of ecosystem services based on remote sensing technology: A sampling of the Gansu grassland ecosystem. Sci. Cold Arid. Reg..

[94] Wei-Dong Y., Feng H., Mei-Bing J., Zeng-Di P., Ye-li Y. (1998). Hydrodynamical Basis for Interpreting the Features of a Kind of Ocean Objects on Synthetic Aperture Radar Images. Chin. Phys. Lett..

[95] Vázquez-González C., Ávila-Foucat V.S., Ortiz-Lozano L., Moreno-Casasola P., Granados-Barba A. (2021). Analytical framework for assessing the social-ecological system trajectory considering the resilience-vulnerability dynamic interaction in the context of disasters. Int. J. Disaster Risk Reduct..

[96] Dakos V., Kefi S. (2022). Ecological resilience: What to measure and how. Environ. Res. Lett..

[97] Qi S. (2017). Correlation between Watershed Ecological Vulnerability and Ecosystem Services: A Case Study of Bailongjiang Watershed in Gansu Province.

[98] Depietri Y., Welle T., Renaud F. (2013). Social vulnerability assessment of the Cologne urban area (Germany) to heat waves: Links to ecosystem services. Int. J. Disaster Risk Reduct..

[99] Cowles J., Templeton L., Battles J.J., Edmunds P.J., Carpenter R.C., Carpenter S.R., Nelson M.P., Cleavitt N.L., Fahey T.J., Groffman P.M. (2021). Resilience: Insights from the U.S. LongTerm Ecological Research Network. Ecosphere.

[100] Xiong K., Chi Y. (2015). Problems and countermeasures facing the karst ecosystem in southern China. Ecol. Econ..

[101] Song S., Xiong K., Chi Y., Shen X., Guo T., Lu N. (2018). Research progress and prospect of grassland establishment and ecological animal husbandry in the karst rocky desertification area. Fresenius Environ. Bull..

[102] Jianhua C., Daoxian Y., LiQiang T., Mallik A., Hui Y., Fen H. (2015). An Overview of Karst Ecosystem in Southwest China: Current State and Future Management. J. Resour. Ecol..

[103] He M. (2019). The Research of Ecosystem Vulnerability in Southwestern China Based on Vegetation Productivity.

[104] Yang Q., Meng G., Gu L., Fang B., Zhang Z., Cai X. (2021). A review on the methods of assessment for the service of grassland ecosystem. Ecol. Sci..

[105] Guo C., Ma W., Zhao C., Li J., Wang Y., Xi Y., Wei S. (2019). The effect of snow soil respiration rate in subalpine meadows of the Qilian Mountains. Acta Ecol. Sinica.

[106] Xiuping Z., Dunjiang S., Shaofeng C. (2019). Ecological Carrying Capacity of Grasslands and Animal Husbandry Sustainability in Central Asia. J. Resour. Ecol..

[107] Geng G. (2018). The expansion trend of rocky desertification land in China has been resersed—The State Forestry and Grassland Administration announces the results of the third rocky desertification monitoring. Green China.

[108] Liu H., Liu L., Ding S. (2017). The impact of human activities on ecosystem services flow. Acta Ecol. Sin..

[109] Lauerburg R., Diekmann R., Blanz B., Gee K., Held H., Kannen A., Möllmann C., Probst W., Rambo H., Cormier R. (2019). Socio-ecological vulnerability to tipping points: A review of empirical approaches and their use for marine management. Sci. Total Environ..

[110] Zhang X., Niu J., Buyantuev A., Zhang Q., Dong J., Kang S. (2016). Understanding Grassland Degradation and Restoration from the Perspective of Ecosystem Services: A Case Study of the Xilin River Basin in Inner Mongolia, China. Sustainability.

[111] Spake R., Lasseur R., Crouzat E., Bullock J.M., Lavorel S., Parks K.E., Schaafsma M., Bennett E.M., Maes J., Mulligan M. (2017). Unpacking ecosystem service bundles: Towards predictive mapping of synergies and trade-offs between ecosystem services. Glob. Environ. Chang..

[112] Lee H., Lautenbach S. (2016). A quantitative review of relationships between ecosystem services. Ecol. Indic..

[113] Agudelo C.A.R., Bustos S.L.H., Moreno C.A.P. (2020). Modeling interactions among multiple ecosystem services. A critical review. Ecol. Model..

[114] Fischer A.P., Paveglio T., Carroll M., Murphy D., Brenkert-Smith H. (2013). Assessing Social Vulnerability to Climate Change in Human Communities near Public Forests and Grasslands: A Framework for Resource Managers and Planners. J. For..

[115] Yang W.S., Liu Y., Zhao J., Chang X., Wu G.L. (2021). Soc changes were more sensitive in alpine grasslands than in temperate grasslands during grassland transformation in china: A meta-analysis. J. Clean. Prod..

[116] Raheem N., Cravens A.E., Cross M.S., Crausbay S., Ramirez A., McEvoy J., Zoanni D., Bathke D.J., Hayes M., Carter S. (2019). Planning for ecological drought: Integrating ecosystem services and vulnerability assessment. J. Wiley Interdiscip. Rev. Water.

